# Exercise Volume Provides New Insight into the Effects of Housing Systems on Chicken Body Conformation, Carcass Traits, Meat Quality, and Serum Biochemical Parameters

**DOI:** 10.3390/ani14162387

**Published:** 2024-08-17

**Authors:** Peng Ren, Li Zhou, Yingfeng Xu, Meiying Chen, Zhengwei Luo, Jingjing Li, Yiping Liu

**Affiliations:** 1 School of Life Science and Engineering, Southwest University of Science and Technology, Mianyang 621010, China; pengren@swust.edu.cn (P.R.); cmy18599950531@163.com (M.C.); m13198691829@163.com (Z.L.); 2 Yibin Academy of Agricultural Sciences, Yibin 644600, China; zhoulirunan@sina.cn; 3 Farm Animal Genetic Resources Exploration and Innovation Key Laboratory of Sichuan Province, Sichuan Agricultural University, Chengdu 611130, China; uyingfeng1@muyuanfoods.com

**Keywords:** chicken, step counts, carcass trait, meat quality, serum biochemistry

## Abstract

**Simple Summary:**

This study investigates how different housing systems affect daily step counts and their impact on body conformation, carcass traits, meat quality, and serum biochemical parameters of Jiuyuan Black chickens. Chickens were monitored from 60 to 150 days old, with step counts recorded using pedometers. The study showed that free-range chickens with high step counts had significantly higher daily and total steps compared to other groups, as well as higher heart weight, lower abdominal fat, and better meat quality, but lower intramuscular fat. The CK and LDH levels in chickens exhibited an almost consistent upward trend as exercise levels increased. Overall, free-range chickens with more exercise volume improved heart weight and reduced fat but negatively impacted some body measurements and meat quality. These findings help in choosing housing systems for Chinese indigenous chickens.

**Abstract:**

This study aims to investigate the dynamic changes in daily step counts under different housing systems and further explore the effects of housing system on the body conformation, carcass traits, meat quality, and serum biochemical parameters of a Chinese indigenous chicken breed. At 60 d of age, 300 Jiuyuan Black male chickens with similar body weights in each housing system were further raised until the age of 150 d. At 90, 120, and 150 d of age, in both cage-reared and free-range systems, the top 20 chickens with the highest step counts measured using pedometers and the bottom 20 chickens with the lowest step counts were designated as the cage high-steps group (CHS), the cage low-steps group (CLS), the free-range high-steps group (FHS), and the free-range low-steps group (FLS), respectively. The results show that, at any age stage, the average daily steps (ADS) and total steps (TS) of the FHS group are significantly higher than the other three groups (*p* < 0.05). The TS of almost all groups showed an overall downward trend as the age increased. Increased exercise volume results in reduced shank length (90 d), breast width (90 d), and keel length (150 d) (*p* < 0.05). Only birds at 90 d of age from the FHS and FLS groups exhibited lower live body weight, carcass weight, half-eviscerated weight, eviscerated weight, breast muscle weight, leg muscle weight, and percentage of eviscerated weight than the CLS group (*p* < 0.05). Birds from the FHS group showed the highest heart weight values but the lowest abdominal fat weight values among these four groups (*p* < 0.05). Both the breast and leg muscle samples from the FHS group displayed higher dry matter and shear force than those from the CHS and CLS groups (*p* < 0.05). The FHS group displayed the lowest intramuscular fat among the four groups (*p* < 0.05). The creatine kinase (CK) and lactate dehydrogenase (LDH) levels in chickens of all age stages were almost observed to rise with increased physical activity. In conclusion, free-range chickens with more exercise volume exhibited an elevated heart weight and reduced abdominal fat but showed negative effects on some body measurements and carcass traits. These results can provide a theoretical basis for the selection of different housing systems for Chinese indigenous chickens.

## 1. Introduction

As one of the most numerous and ubiquitous domestic animals, chickens (*Gallus gallus domesticus*) provide abundant, high-quality, and affordable meat and egg protein for humans [1]. Numerous studies have demonstrated that housing systems can impact the welfare, physiology, health, productivity, and product quality of poultry by modifying their physical activity, such as through the provision of perches, addition of ramps, implementation of barriers between food and water, or changing the rearing system [2,3]. Due to variations in factors such as species, age, nutrition, density, etc., the effects of different housing systems on poultry performance and product quality indicators remain controversial.

Recently, Guo et al. (2019) and Wang et al. (2021) used pedometers to track and measure the walking steps of chickens in a free-range setting to assess the effect of exercise on growth, carcass yield, meat quality, muscle mRNA expression profiles, and tibial strength [4,5]. Their findings provide fresh perspectives on the effects of exercise on chickens, indicating that increased exercise led to higher body weight, enhanced meat quality through increased water-holding capacity and reduced shear force values, decreased percentage of breast muscle weight, and improved tibial strength. However, the differences in exercise profiles among birds under different rearing systems and the specific effect of housing systems on poultry from the perspective of variations in physical activity levels remain largely unknown.

Previous reports have demonstrated that slow-growing poultry breeds are the ones that can derive maximum benefits from a free-range housing system, as fast-growing birds exhibit a significantly lower level of adaptability [6,7,8,9]. Traditionally, Chinese indigenous chickens are selected not primarily for the meat yield capacity but for organic qualities, high meat quality, and intense flavor [10]. Therefore, Jiuyuan Black chickens, a typical slow-growing breed extensively raised in southwestern China, were selected for this study.

In the present study, a pedometer was used to record the daily step counts of Jiuyuan Black chickens under different housing systems. The main purpose of this study was to investigate the effects of different housing systems on the body conformation, carcass traits, meat quality, and serum biochemical parameters of slow-growing chickens from the perspective of differences in exercise levels.

## 2. Materials and Methods

### 2.1. Animals and Experimental Design

In this study, 30-day-old Jiuyuan Black male chickens (a Chinese indigenous chicken breed) were randomly housed in conventional cages and a free-range environment reared with 1500 individuals each. At an age ranging from 30 to 60 days, the chickens were housed in triple-layered, three-dimensional cages of 70 cm × 70 cm × 40 cm (length × width × height) with a density of 10 birds per cage. At 60 d of age, 300 chickens with similar body weights in each housing system were further randomly divided into 3 groups of 100 individuals each: from 60 to 90 d of age, from 90 to 120 d of age, and from 120 to 150 d of age, and the stocking density was adjusted to 1 cage per bird. In the free-range model, chickens were maintained in the hills at a density of 150 birds per 667 m^2^. The daily step counts of all experimental chickens in each group were recorded individually by an intelligent pedometer system consisting of a smart ankle bracelet (pedometer), a signal receiving device, and a data storage system (Shenzhen Lianwen Intelligent Technology Co., Ltd., Shenzhen, China). The daily step counts of all experimental chickens in each group were recorded by the pedometer (Shenzhen Lianwen Intelligent Technology Co., Ltd., Shenzhen, China). Based on the average daily step counts, the chickens of each group mentioned above were further chosen as the high-steps group (the top 20 birds) and low-steps group (the last 20 birds). Therefore, based on the difference of the housing system and exercise volume, this study was finally divided into four experimental groups: the cage high-steps group (CHS), the cage low-steps group (CLS), the free-range high-steps group (FHS), and the free-range low-steps group (FLS). All birds were raised with free access to feed and water in the Poultry Breeding Farm of Wan yuan Hengkang Agricultural Development Co., Ltd., Wanyuan, China.

### 2.2. Sample Collection and Measurement

At 90, 120, and 150 d of age, 20 chickens of each experimental group were randomly selected for live body conformation measurement after a 12 h fast. After the individual live body weight (LBW) was recorded, live body conformation measurements, which included body slope length (BSL, the body surface distance from shoulder joint to ischial tuberosity), keel length (KL, the body surface distance from the front to the end of the keel), breast width (BW, the distance between two shoulder joints), breast depth (BD, the distance from the first thoracic vertebra to the front of the keel), and shank length (SL, the distance from the hind corner of the hock joint to the first scale of the third toe), were measured with a digital vernier caliper and flexible measuring tape [5,11,12]. To avoid individual variations in measurements, all the indicators above were measured by the same trained person.

In addition, 15 birds of each experimental group were randomly selected, and blood samples were collected from the wing vein. The blood collection tubes were centrifuged at 3000 rpm for 15 min to separate the serum [13], and the serum samples were stored at −20 °C until further use. The levels of lactate dehydrogenase (LDH) and creatine kinase (CK) in the serum were detected by using the enzyme-linked immunosorbent assay (ELISA) kit, following the manufacturer’s instructions (Enzyme-linked Biotechnology Co., Ltd., Shanghai, China).

The above 20 birds of each experimental group with measured live body conformation were electrically stunned and killed by exsanguination, and then scalded, defeathered, weighed (carcass weight, CW), eviscerated, and divided. The dressing percentage was calculated as the ratio between CW and LBW. Carcass traits were measured using the method called performance terms and measurement for poultry by the Agricultural Ministry of China (NY/T 823-2004 [14]) as follows:

Half-eviscerated weight (HEW) refers to the carcass after subtracting the weight of the trachea, esophagus, full crop, intestines, spleen, pancreas, gallbladder, and reproductive organs. Eviscerated weight (EW) was calculated by subtracting the weight of the heart, liver, glandular stomach, gizzard, lung, abdominal fat, head, and legs from the half-eviscerated weight. The proportion of the HEW and EW in the LBW, the percentage of the leg muscle and breast muscle of the EW, and the percentage of abdominal fat of the weight (EW + abdominal fat weight) were calculated, respectively. Additionally, heart weight (HW), liver weight (LW), glandular stomach weight (GSW), gizzard weight (GW), and abdominal fat weight (AFW) were measured. The percentages of heart weight (PHW), liver weight (PLW), glandular stomach weight (PGSW), gizzard weight (PGW), and abdominal fat weight (PAFW) were calculated, respectively.

After the carcass parameters were determined, the breast and leg muscle samples of all individuals above were further collected for meat quality analyses. The pH values were measured at 24 h (pH_24h_) postmortem (stored at 4 °C) at 1 cm depth in triplicate using a pH-Star meter (Orion Co., Ltd., Boston, MA, USA). By hanging the whole piece of meat (1.0 cm × 1.0 cm × 2.0 cm) in a sealed pocket at 4 °C for 24 h, drip loss was calculated as the percentage of weight loss during storage. Muscle shear force was measured by using the C-LM3 digital meat tenderness meter (North-east Agricultural University, Harbin, China) with the methods described by [4]. Dry matter (DM) was measured by oven drying, and intramuscular fat (IMF) was determined using Soxhlet petroleum–ether extraction [15]. The content of inosinic acid (IMP) was measured by Agilent 1100 High-Performance Liquid Chromatography (Agilent, Co., Ltd., Palo Alto, CA, USA).

### 2.3. Statistical Analysis

All values were expressed as the means ± standard deviation (SD), and statistical significance was analyzed using a one-way analysis of variance (ANOVA) followed by Duncan’s multiple range analysis by the SPSS version 19.0 software (SPSS Inc., Chicago, IL, USA). Differences were considered significant at *p* < 0.05.

## 3. Results

### 3.1. Exercise Volume Statistics

**Figure 1:**
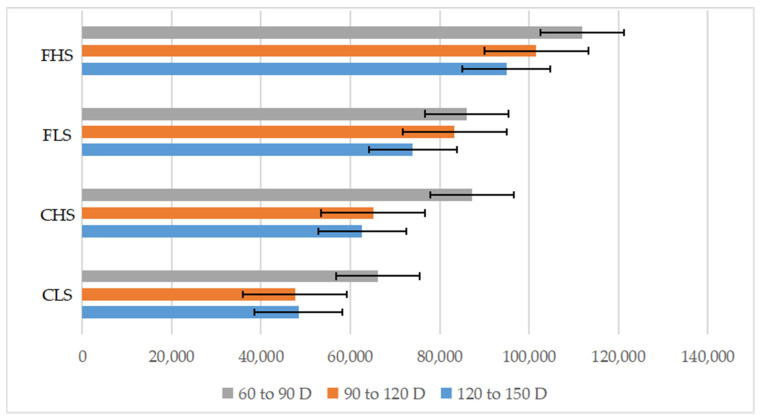
Trends of total steps among different groups of different age periods.

The exercise volume of Jiuyuan Black male chickens in each experimental group is shown in Table 1. No matter what kind of age period (60 to 90 d, 90 to 120 d, or 120 to 150 d), the chickens in the FHS groups showed higher average daily steps (ADS) and total steps (TS) than those in the CHS groups (*p* < 0.05), and birds in the FLS groups showed higher ADS and TS than those in the CLS groups (*p* < 0.05). Interestingly, we found that the TS of almost all experimental groups showed an overall downward trend as the age increased (Figure 1).

### 3.2. Body Conformation

Body conformation values among the CHS, CLS, FHS, and FLS groups at 90 d, 120 d, and 150 d of age are presented in Table 2. There was no significant influence of different experimental groups on the BSL and BD at 90 d, 120 d, and 150 d of age (*p* > 0.05). At 90 d of age, the CLS birds had wider breasts than CHS, FHS, and FLS (*p* < 0.05), and CLS birds showed longer shanks than FHS (*p* < 0.05). At 120 d of age, the CLS birds had narrower breasts than CHS and FHS (*p* < 0.05). At 150 d of age, the CLS birds had longer keels than FHS and FLS (*p* < 0.05).

### 3.3. Carcass Traits

Carcass yield data for the birds are presented in Table 3 and Table 4. At 90 d of age, the LBW, CW, HEW, EW, BMW, LMW, and PEW values of the CLS chickens were higher than those of the FHS and FLS groups (*p* < 0.05). At 120 d of age, the FLS birds showed lower LBW values than those of CHS and CLS (*p* < 0.05), while DP and PHEW values were significantly higher (*p* < 0.05). At 150 d of age, the CLS birds had higher PHEW and PEW values than FHS group (*p* < 0.05). However, there was no significant difference in the PBMW and PLMW values of the 4 groups at 90, 120, and 150 d of age (*p* > 0.05). In addition, the FHS group displayed a higher HW value than the CHS and CLS groups at 90 d of age (*p* < 0.05). Compared to the CHS group, the FHS and FLS groups showed higher LW values at 120 d of age (*p* < 0.05). Moreover, the FHS and FLS groups exhibited lower AFW values than the CLS group at 90, 120, and 150 d of age (*p* < 0.05).

### 3.4. Meat Quality

The meat quality characteristics of the breast and leg muscles are shown on the left and right side of Table 5, respectively. The pH_24_ of CLS group at 120 d of the leg muscle was higher than the others (*p* < 0.05). The CHS and FHS groups displayed a higher dry matter than the CLS and FLS groups at 90 d of the breast and leg muscles (*p* < 0.05). At 120 d of age, the FHS birds showed a higher dry matter than those of FLS in the breast muscle (*p* < 0.05), while the CHS birds showed a higher dry matter than those of CLS in the leg muscle (*p* < 0.05). At 150 d of age, the CHS birds had a higher dry matter than those of the FLS group in the breast muscle, while the FHS birds showed a higher dry matter than those of the CLS and FLS groups in the leg muscle (*p* < 0.05). All the CHS groups showed the least shear force, and the values of CHS groups were significantly lower than those of the FHS and FLS groups at 90, 120, and 150 d of age (*p* < 0.05). Almost all the FHS groups displayed the least intramuscular fat. At 90 d of age, the FHS birds showed lower intramuscular fat than those of CHS and CLS in the breast muscle (*p* < 0.05), while the FHS and FLS birds showed lower intramuscular fat than those of CLS in the leg muscle (*p* < 0.05). At 120 d of age, the FHS birds showed lower intramuscular fat than those of CHS in the breast muscle (*p* < 0.05), while the FHS and FLS birds showed lower intramuscular fat than those of CHS in the leg muscle (*p* < 0.05). However, no significant differences were noted in the drip loss and inosine monophosphate among the four groups in the breast and leg muscles (*p* > 0.05).

### 3.5. Serum Biochemical Parameters

The serum biochemical parameters of CK and LDH are shown in Table 6. Regardless of the time point, the overall serum CK and LDH levels in the CHS and CLS groups were higher than those in the FHS and FLS groups. Specifically, no significant difference was observed in serum CK content among groups at 90 d of age (*p* > 0.05), whereas the CHS group displayed higher serum LDH level than the CLS, FHS, and FLS groups (*p* < 0.05). At 120 d of age, the birds in the CHS and CLS groups had a higher serum CK content than those in the FHS and FLS groups (*p* < 0.05), while the CHS birds showed a higher serum LDH level than those of the CLS, FHS, and FLS groups (*p* < 0.05). At 150 d of age, the CHS birds had a higher serum CK content than those of the FLS group (*p* < 0.05), while the CHS birds showed a higher serum LDH level than those of the FHS group (*p* < 0.05).

## 4. Discussion

The results show that the ADS or TS of all birds decreased from 60 d to 150 d. This result is inconsistent with that of Wang et al. (2021), who reported that sex and environmental factors, but not age, could affect the amount of exercise during the adolescent stage of Chinese indigenous chickens [5]. The reasons for this discrepancy could be attributed to the differences of breed and test period. Clearly, the TS of the CHS and CLS groups were significantly lower than those in the FHS and FLS groups with the increase in age (from D60 to D90), which implied that the reduction in the amount of exercise in cage birds may be affected by the continuous decrease in activity space. Compared to traditional studies on the effects of different feeding models on animal welfare and production performance [16,17,18], this research offers a new perspective for similar studies. The findings indicate that feeding models, age, and individual differences may influence the amount of exercise observed in chickens.

The present study showed that increased exercise volume results in reduced shank length (90 d), breast width (90 d), and keel length (150 d), which suggested that increased exercise may have a negative impact on partial body measurements. This is partly in accordance with the finding of Wang et al. (2021), who reported that birds with a higher exercise volume showed a shorter shank length [5]. Additionally, previous studies showed that chicken reared in a free-range system or with a high number of step counts displayed a higher shank strength, bone density, and total bone mineral content than birds from conventional cages [5,19], which implied that increased exercise volume or free-range systems could have positive effects on the chicken bone characteristics.

In the present study, only birds at 90 d of age from the FHS and FLS groups exhibited lower live body weight, carcass weight, half-eviscerated weight, eviscerated weight, breast muscle weight, leg muscle weight, and percentage of eviscerated weight than the CLS group, inconsistent with previous studies [5,20,21], and indicated that no difference observed in the carcass and parts yields between conventional and free-range or high exercise group and low exercise group. The indigenous chicken breed selected in this study is still not at somatic maturity at 90 d of age, and the difference in exercise volume caused by the housing systems and individual differences at this age stage may lead to this phenomenon. Interestingly, birds from the FHS group showed the highest heart weight values but the lowest abdominal fat weight values among these four groups. Our study suggested that increased exercise can effectively increase heart weight and reduce abdominal fat in poultry, which were consistent with previous studies [5,20,22].

The breeds that are indigenous to China are not primarily selected for their meat yield capacity but for organic purposes, high meat quality, and intense flavor [10]. Both the dry matter and shear force are important meat quality characteristics. The dry matter refers to everything in the meat, except for the water content. Shear force is an important parameter of tenderness, with lower values indicating greater tenderness [23,24]. The present study showed that both the breast and leg muscle samples from the FHS group displayed a higher dry matter and shear force than those from the CHS and CLS groups, and similar findings have been reported by Castellini et al. (2002), who demonstrated that the housing system affected the shear force that was higher in either the breast or leg of the organic birds [7]. In addition, Jin et al. (2019) reported that shear force increased linearly with the increase in free-range days [25]. These findings might be due to the increased motor activity under the free-range system, which could contribute to development of the skeletal muscle, and thus might affect the shear force. However, Guo et al. (2019) and Wang et al. (2021) stated that increased exercise decreased the shear force [4,5], and Wang et al. (2009) reported that the raising system had no effect on the shear force [20]. These discrepancies may be due to differences in breed, age, environmental factors, and farming systems. It is important to acknowledge that, out of consideration for animal welfare, consumers in certain countries may be willing to accept a slightly lower quality in free-range systems. Intramuscular fat is another important evaluation index of meat quality, which is positively correlated with tenderness and flavor [26,27]. Our data indicated that the FHS group displayed the lowest intramuscular fat among the four groups, which showed a similar trend to the abdominal fat weight results. Similarly, previous studies indicated that non-caged chickens showed thinner subcutaneous fat and lower abdominal fat and intramuscular fat contents [28,29]. The increased activity of chickens in the free-range system may be a contributing factor to this phenomenon, resulting in a greater dietary energy consumption and correspondingly less fat deposition.

In 1958, the first reported increase in serum enzyme activity following exercise in humans marked the beginning of subsequent studies that have established the multifactorial nature of the degree to which the serum activities of various enzymes increase during and after exercise [30]. Increased serum concentrations of creatine kinase (CK) and lactate dehydrogenase (LDH) are used as indicators of damage to muscle membrane and other tissue structures, as these molecules are fragments of the myosin heavy chain and are unable to cross the sarcoplasmic membrane barrier [31,32,33]. Generally, the serum CK concentration has been considered to be the best indirect index of human skeletal muscle damage, particularly for the diagnosis of some diseases [34], and showed large variability between subjects [35]. Moreover, both CK, closely associated with intracellular energy metabolism and muscle contraction, and LDH, responsible for catalyzing the interconversion of pyruvate and lactate, have been shown to physiologically increase in humans with rising levels of physical activity [36,37,38]. Interestingly, our study indicates that the CK and LDH levels in chickens of all age stages almost have been observed to rise with increased physical activity, which suggests that serum CK and LDH levels can be used to indirectly reflect the actual level of physical activity in chickens.

## 5. Conclusions

In summary, the present study indicated that feeding models, age, and individual differences could affect the level of exercise in chickens. The FHS group exhibited elevated heart weight and reduced abdominal fat but showed negative effects on some body measurements and carcass traits. Moreover, increased exercise impacted the meat quality of breast and leg muscles by increasing the dry matter content, while decreasing the intramuscular fat. Furthermore, as exercise levels increased, chicken CK and LDH levels showed an almost consistent upward trend.

## Figures and Tables

**Table 1:** Comparison of the average and total steps among different groups.

Item	CHS ^1^	CLS ^1^	FHS ^1^	FLS ^1^
60 to 90 d				
^2^ ADS	2908.01 ± 540.66 ^b^	2141.22 ± 61.04 ^c^	3730.22 ± 499.96 ^a^	2868.59 ± 250.11 ^b^
^2^ TS	83,495.63 ± 15,214.63 ^b^	66,242.86 ± 4285.00 ^c^	111,907 ± 14,998.85 ^a^	86,057.71 ± 7503.18 ^b^
90 to 120 d				
^2^ ADS	2170.27 ± 284.22 ^c^	1586.53 ± 211.66 ^d^	3385.60 ± 335.86 ^a^	2775.81 ± 104.30 ^b^
^2^ TS	65,108.00 ± 8526.49 ^c^	47,596.00 ± 6349.83 ^d^	101,568.00 ± 10,075.84 ^a^	83,274.29 ± 3129.02 ^b^
120 to 150 d				
^2^ ADS	2086.15 ± 114.36 ^c^	1611.91 ± 233.76 ^d^	3164.00 ± 501.07 ^a^	2465.65 ± 113.2 ^b^
^2^ TS	62,584.33 ± 3430.69 ^c^	48,357.33 ± 7012.79 ^d^	94,920.20 ± 15,032.10 ^a^	73,969.40 ± 3396.35 ^b^

Values are expressed as means ± standard deviation (SD). Means within a row marked without the same superscripts differed significantly (*p* < 0.05). ^1^ CHS, the cage high-steps group; CLS, the cage low-steps group; FHS, the free-range high-steps group; FLS, the free-range low-steps group. ^2^ ADS, average daily steps; TS, total steps.

**Table 2:** Comparison of body measurements among different groups.

Item	CHS ^1^	CLS ^1^	FHS ^1^	FLS ^1^
^2^ BSL (cm)				
90 d	20.95 ± 0.67	21.74 ± 0.78	21.00 ± 0.98	21.36 ± 1.31
120 d	22.57 ± 1.28	22.37 ± 1.10	22.6 ± 0.99	22.63 ± 1.19
150 d	23.45 ± 0.69	23.65 ± 0.41	23.45 ± 0.95	23.81 ± 1.04
^2^ KL (cm)				
90 d	10.83 ± 0.85	11.43 ± 1.11	10.68 ± 0.61	10.84 ± 0.42
120 d	10.91 ± 1.12	10.83 ± 1.69	11.63 ± 0.69	11.6 ± 0.80
150 d	12.73 ± 0.27 ^ab^	13.21 ± 0.46 ^a^	12.22 ± 1.00 ^b^	12.33 ± 0.69 ^b^
^2^ BW (cm)				
90 d	7.00 ± 0.35 ^b^	8.12 ± 0.84 ^a^	7.04 ± 0.15 ^b^	7.02 ± 0.43 ^b^
120 d	7.90 ± 0.34 ^a^	7.44 ± 0.27 ^b^	7.89 ± 0.29 ^a^	7.79 ± 0.45 ^ab^
150 d	7.61 ± 0.50	8.05 ± 0.62	7.88 ± 0.30	8.08 ± 0.24
^2^ BD (cm)				
90 d	11.46 ± 0.20	11.44 ± 0.32	10.63 ± 0.84	11.25 ± 0.52
120 d	12.52 ± 0.56	12.33 ± 0.54	12.77 ± 0.19	12.34 ± 0.69
150 d	12.10 ± 0.74	12.39 ± 0.30	12.01 ± 0.28	12.20 ± 0.46
^2^ SL (cm)				
90 d	9.39 ± 0.33 ^ab^	9.89 ± 0.22 ^a^	9.13 ± 0.18 ^b^	9.42 ± 0.56 ^ab^
120 d	10.44 ± 0.82	9.89 ± 0.52	10.41 ± 0.52	10.18 ± 0.57
150 d	10.34 ± 0.86	10.11 ± 0.24	9.68 ± 0.55	10.32 ± 0.66

Values are expressed as means ± SD. Means within a row marked without the same superscripts differed significantly (*p* < 0.05). ^1^ CHS, the cage high-steps group; CLS, the cage low-steps group; FHS, the free-range high-steps group; FLS, the free-range low-steps group. ^2^ BSL, body slope length; KL, keel length; BW, breast width; BD, breast depth; SL, shank length.

**Table 3:** Comparison of slaughtering performance among different groups.

Item	CHS ^1^	CLS ^1^	FHS ^1^	FLS ^1^
^2^ LBW (g)				
90 d	1562.5 ± 57.52 ^b^	1735.00 ± 161.34 ^a^	1402.00 ± 91.83 ^bc^	1385.00 ± 132.90 ^c^
120 d	1979.86 ± 183.33 ^a^	1985.00 ± 140.21 ^a^	1892.86 ± 191.50 ^ab^	1759.29 ± 151.67 ^b^
150 d	2063 ± 118.24	2199 ± 120.15	2128 ± 114.54	2237 ± 216.12
^2^ CW (g)				
90 d	1440.00 ± 70.36 ^ab^	1590.00 ± 140.06 ^a^	1313.00 ± 66.48 ^b^	1310.90 ± 132.00 ^b^
120 d	1651.43 ± 152.30	1625.29 ± 109.61	1615.71 ± 211.03	1600 ± 121.93
150 d	1760 ± 134.77	1884 ± 111.21	1799 ± 115.46	1888 ± 193.80
^2^ HEW (g)				
90 d	1265.00 ± 57.15 ^ab^	1416.5 ± 129.12 ^a^	1134 ± 96.85 ^b^	1126.00 ± 144.59 ^b^
120 d	1520.00 ± 161.04	1485.71 ± 119.11	1503.57 ± 180.72	1402.14 ± 144.54
150 d	1625 ± 120.68	1766 ± 102.13	1638 ± 113.72	1755 ± 186.15
^2^ EW (g)				
90 d	1052.50 ± 43.30 ^a^	1178.75 ± 116.29 ^a^	911.00 ± 63.48 ^b^	920.00 ± 119.22 ^b^
120 d	1274.29 ± 127.75	1751.43 ± 112.24	1236.43 ± 166.83	1158.57 ± 121.54
150 d	1368 ± 118.56	1506 ± 93.90	1394 ± 96.66	1489 ± 163.87
^2^ BMW (g)				
90 d	148.7 ± 31.37 ^ab^	170 ± 27.73 ^a^	124.28 ± 37.31 ^b^	123.28 ± 37.31 ^b^
120 d	181.26 ± 33.71	180.31 ± 24.29	171.69 ± 31.81	174.91 ± 26.34
150 d	199 ± 27.63	229 ± 39.29	220.72 ± 19.05	234.88 ± 34.10
^2^ LMW (g)				
90 d	233.85 ± 12.68 ^ab^	255.70 ± 27.76 ^a^	201.32 ± 17.39 ^b^	206.16 ± 30.81 ^b^
120 d	297.74 ± 33.04	289.09 ± 42.71	290.51 ± 47.18	278.40 ± 44.55
150 d	375.28 ± 95.41	409.16 ± 24.40	376.76 ± 44.63	400.20 ± 48.30
^2^ DP (%)				
90 d	92.14 ± 1.67	91.68 ± 0.85	93.73 ± 1.54	94.57 ± 2.40
120 d	83.21 ± 1.38 ^b^	81.90 ± 1.14 ^b^	85.24 ± 7.37 ^ab^	88.89 ± 2.21 ^a^
150 d	85.24 ± 1.99	86.12 ± 1.28	84.50 ± 1.15	84.36 ± 0.84
^2^ PHEW (%)				
90 d	80.94 ± 0.86	81.65 ± 0.80	80.85 ± 3.08	81.13 ± 4.06
120 d	76.51 ± 1.44 ^b^	74.81 ± 1.58 ^b^	79.34 ± 2.68 ^a^	79.61 ± 2.05 ^a^
150 d	78.71 ± 1.68 ^ab^	80.30 ± 1.13 ^a^	76.93 ± 1.51 ^b^	78.41 ± 1.58 ^ab^
^2^ PEW (%)				
90 d	67.35 ± 0.46 ^a^	67.91 ± 1.09 ^a^	64.97 ± 1.09 ^b^	64.91 ± 1.66 ^b^
120 d	64.19 ± 1.73	62.99 ± 2.05	65.17 ± 3.24	65.77 ± 1.67
150 d	66.23 ± 2.45 ^ab^	68.47 ± 1.61 ^a^	65.48 ± 1.87 ^b^	66.50 ± 1.40 ^ab^
^2^ PBMW (%)				
90 d	14.06 ± 2.41	14.35 ± 1.01	13.70 ± 1.89	14.56 ± 2.21
120 d	14.20 ± 1.94	14.37 ± 0.93	13.86 ± 1.45	15.07 ± 1.31
150 d	14.53 ± 1.39	15.28 ± 2.84	15.85 ± 1.16	15.75 ± 1.22
^2^ PLMW (%)				
90 d	22.21 ± 0.52	21.72 ± 1.55	22.09 ± 1.00	22.85 ± 1.72
120 d	23.42 ± 2.15	23.02 ± 1.91	23.45 ± 1.51	23.94 ± 1.76
150 d	27.19 ± 5.52	27.19 ± 0.91	27.07 ± 3.20	27.07 ± 3.85

Values are expressed as means ± SD. Means within a row marked without the same superscripts differed significantly (*p* < 0.05). ^1^ CHS, the cage high-steps group; CLS, the cage low-steps group; FHS, the free-range high-steps group; FLS, the free-range low-steps group. ^2^ LBW, live body weight; CW, carcass weight; HEW, half-eviscerated weight; EW, eviscerated weight; BMW, breast muscle weight; LMW, leg muscle weight; DP, dressing percentage; PHEW, percentage of half-eviscerated weight; PEW, percentage of eviscerated weight; PBMW, percentage of breast muscle weight; PLMW, percentage of leg muscle weight.

**Table 4:** Comparison of visceral organ weights among different groups.

Item	CHS ^1^	CLS ^1^	FHS ^1^	FLS ^1^
^2^ HW (g)				
90 d	6.70 ± 0.75 ^b^	6.55 ± 0.50 ^b^	8.13 ± 0.49 ^a^	7.43 ± 0.84 ^ab^
120 d	8.07 ± 1.11	8.58 ± 1.14	8.98 ± 1.84	8.68 ± 2.21
150 d	9.55 ± 0.90	9.45 ± 0.75	10.73 ± 2.00	10.67 ± 1.92
^2^ LW (g)				
90 d	28.43 ± 4.52	32.76 ± 2.62	33.7 ± 3.83	32.8 ± 4.70
120 d	30.45 ± 4.19 ^b^	34.40 ± 6.48 ^ab^	39.10 ± 4.62 ^a^	40.92 ± 5.17 ^a^
150 d	29.17 ± 4.15	31.60 ± 4.30	38.12 ± 11.16	39.57 ± 5.40
^2^ GSW (g)				
90 d	5.70 ± 0.47	5.75 ± 0.48	4.56 ± 0.57	5.55 ± 0.10
120 d	5.30 ± 0.91	5.83 ± 0.70	6.08 ± 1.15	5.13 ± 2.77
150 d	5.15 ± 0.89	5.2 ± 0.55	5.05 ± 1.01	5.05 ± 0.91
^2^ GW (g)				
90 d	26.90 ± 5.74	22.62 ± 3.05	25.95 ± 6.72	24.78 ± 3.38
120 d	29.65 ± 6.11	29.93 ± 5.15	30.08 ± 2.84	30.32 ± 7.27
150 d	25.50 ± 2.91	26.67 ± 4.52	24.35 ± 6.23	24.40 ± 2.58
^2^ AFW (g)				
90 d	8.78 ± 5.85 ^a^	10.92 ± 10.40 ^a^	2.125 ± 2.14 ^b^	3.33 ± 1.93 ^b^
120 d	3.37 ± 2.97 ^a^	4.22 ± 2.30 ^a^	2.02 ± 2.59 ^b^	2.68 ± 2.32 ^b^
150 d	4.67 ± 1.08 ^ab^	6.05 ± 2.44 ^a^	1.58 ± 2.34 ^c^	2.37 ± 1.09 ^bc^

Values are expressed as means ± SD. Means within a row marked without the same superscripts differed significantly (*p* < 0.05). ^1^ CHS, the cage high-steps group; CLS, the cage low-steps group; FHS, the free-range high-steps group; FLS, the free-range low-steps group. ^2^ HW, heart weight; LW, liver weight; GSW, glandular stomach weight; GW, gizzard weight; AFW, abdominal fat weight.

**Table 5:** Comparison of meat characteristics among different groups.

Item	Breast Muscle				Item	Leg Muscle			
	CHS ^1^	CLS ^1^	FHS ^1^	FLS ^1^		CHS ^1^	CLS ^1^	FHS ^1^	FLS ^1^
^2^ pH_24_					^2^ pH_24_				
90 d	5.75 ± 0.06	5.83 ± 0.09	5.76 ± 0.09	5.77 ± 0.04	90 d	5.85 ± 0.06	5.83 ± 0.05	5.89 ± 0.04	5.87 ± 0.06
120 d	5.99 ± 0.03	6.04 ± 0.06	6.02 ± 0.08	6.06 ± 0.10	120 d	6.10 ± 0.06 ^b^	6.22 ± 0.07 ^a^	6.10 ± 0.14 ^b^	6.07 ± 0.03 ^b^
150 d	6.23 ± 0.11	6.27 ± 0.13	6.11 ± 0.06	6.20 ± 0.13	150 d	6.43 ± 0.08	6.44 ± 0.12	6.35 ± 0.05	6.45 ± 0.08
^2^ DM (%)					^2^ DM (%)				
90 d	33.21 ± 0.27 ^a^	30.22 ± 0.34 ^b^	34.42 ± 0.17 ^a^	30.88 ± 0.31 ^b^	90 d	31.68 ± 0.43 ^a^	30.07 ± 0.80 ^b^	31.59 ± 0.35 ^a^	30.01 ± 1.09 ^b^
120 d	30.23 ± 0.98 ^ab^	29.98 ± 0.11 ^ab^	31.56 ± 0.74 ^a^	29.02 ± 0.52 ^b^	120 d	29.10 ± 0.24 ^a^	27.65 ± 0.34 ^b^	28.76 ± 0.61 ^a^	28.35 ± 0.93 ^ab^
150 d	27.73 ± 0.71 ^a^	27.31 ± 0.45 ^ab^	27.42 ± 0.55 ^ab^	26.59 ± 0.48 ^b^	150 d	23.89 ± 0.64 ^ab^	23.30 ± 0.40 ^b^	24.69 ± 0.91 ^a^	23.18 ± 0.81 ^b^
^2^ SF (Kg)					^2^ SF (Kg)				
90 d	1.79 ± 0.36 ^b^	2.82 ± 0.50 ^a^	3.17 ± 0.39 ^a^	3.65 ± 0.79 ^a^	90 d	2.54 ± 0.38 ^b^	3.25 ± 0.30 ^ab^	3.82 ± 0.23 ^a^	3.81 ± 0.86 ^a^
120 d	2.19 ± 0.63 ^b^	2.66 ± 0.34 ^b^	3.79 ± 0.56 ^a^	3.52 ± 0.37 ^a^	120 d	3.05 ± 0.60 ^b^	3.12 ± 0.39 ^b^	4.37 ± 0.40 ^a^	4.33 ± 0.55 ^a^
150 d	3.51 ± 0.24 ^c^	4.03 ± 0.41 ^bc^	4.65 ± 0.53 ^b^	5.25 ± 0.28 ^a^	150 d	4.12 ± 0.40 ^b^	4.25 ± 0.31 ^b^	4.88 ± 0.47 ^a^	5.32 ± 0.49 ^a^
^2^ DL (%)					^2^ DL (%)				
90 d	0.41 ± 0.90	0.41 ± 0.83	0.38 ± 0.10	0.37 ± 0.89	90 d	0.31 ± 0.77	0.32 ± 0.45	0.32 ± 0.77	0.30 ± 0.06
120 d	0.39 ± 0.03	0.38 ± 0.03	0.31 ± 0.04	0.35 ± 0.06	120 d	0.28 ± 0.04	0.31 ± 0.03	0.29 ± 0.04	0.28 ± 0.05
150 d	0.24 ± 0.72	0.23 ± 0.09	0.24 ± 0.57	0.25 ± 0.65	150 d	0.21 ± 0.43	0.24 ± 0.06	0.22 ± 0.04	0.23 ± 0.36
^2^ IMF (%)					^2^ IMF (%)				
90 d	6.56 ± 0.45 ^a^	6.76 ± 0.64 ^a^	5.5 ± 0.36 ^b^	6.34 ± 0.58 ^ab^	90 d	9.42 ± 0.72 ^ab^	10.56 ± 0.73 ^a^	8.68 ± 0.75 ^b^	8.48 ± 0.79 ^b^
120 d	6.00 ± 0.39 ^a^	5.65 ± 0.30 ^ab^	5.1 ± 0.30 ^b^	5.65 ± 0.45 ^ab^	120 d	9.08 ± 1.16 ^a^	9.97 ± 0.55 ^ab^	8.17 ± 0.56 ^b^	8.12 ± 0.63 ^b^
150 d	4.81 ± 0.91	4.44 ± 0.71	4.2 ± 1.00	4.26 ± 0.55	150 d	7.73 ± 0.62	8.48 ± 1.06	6.81 ± 0.48	6.95 ± 1.35
^2^ IMP (mg/g)					^2^ IMP (mg/g)				
90 d	1.22 ± 0.84	1.21 ± 0.09	1.35 ± 0.59	1.32 ± 0.04	90 d	1.20 ± 0.09	1.16 ± 0.12	1.32 ± 0.06	1.27 ± 0.11
120 d	1.58 ± 0.03	1.56 ± 0.03	1.74 ± 0.52	1.77 ± 0.02	120 d	1.64 ± 0.02	1.67 ± 0.03	1.77 ± 0.04	1.77 ± 0.14
150 d	1.70 ± 0.04	1.69 ± 0.05	1.85 ± 0.12	1.78 ± 0.05	150 d	1.72 ± 0.04	1.76 ± 0.01	1.95 ± 0.36	1.85 ± 0.08

Values are shown as means ± SD. Means within a row marked without the same superscripts differed significantly (*p* < 0.05). ^1^ CHS, the cage high-steps group; CLS, the cage low-steps group; FHS, the free-range high-steps group; FLS, the free-range low-steps group. ^2^ pH_24_, pH at 24 h postmortem; DM, dry matter; SF, shear force; DL, drip loss; IMF, intramuscular fat; IMP, inosine monophosphate.

**Table 6:** Comparison of CK and LD among different groups.

Item		CHS ^1^	CLS ^1^	FHS ^1^	FLS ^1^
^2^ CK (U/mL)					
	90 d	1.17 ± 0.20	1.41 ± 0.10	1.11 ± 0.18	1.14 ± 0.19
	120 d	3.46 ± 0.14 ^a^	2.88 ± 0.34 ^a^	1.60 ± 0.35 ^b^	1.48 ± 0.40 ^b^
	150 d	2.65 ± 0.12 ^a^	2.36 ± 0.15 ^ab^	2.21 ± 0.36 ^ab^	1.86 ± 0.48 ^b^
^2^ LDH (U/mL)					
	90 d	8092.81 ± 233.29 ^a^	7212.52 ± 162.89 ^b^	6992.83 ± 569.57 ^b^	6036.89 ± 160.45 ^b^
	120 d	8841.67 ± 493.19 ^a^	7421.96 ± 356.51 ^b^	5811.23 ± 379.15 ^c^	6875.00 ± 333.75 ^b^
	150 d	8609.69 ± 179.74 ^a^	8127.77 ± 242.48 ^ab^	7610.53 ± 615.43 ^b^	7904.85 ± 413.77 ^ab^

Values are expressed as means ± SD. Means within a row marked without the same superscripts differed significantly (*p* < 0.05). ^1^ CHS, the cage high-steps group; CLS, the cage low-steps group; FHS, the free-range high-steps group; FLS, the free-range low-steps group. ^2^ CK, creatine kinase; LDH, lactate dehydrogenase.

## Data Availability

Data will be available upon request from the corresponding author.
